# Inhibition of ethylene production by putrescine alleviates aluminium-induced root inhibition in wheat plants

**DOI:** 10.1038/srep18888

**Published:** 2016-01-08

**Authors:** Yan Yu, Chongwei Jin, Chengliang Sun, Jinghong Wang, Yiquan Ye, Weiwei Zhou, Lingli Lu, Xianyong Lin

**Affiliations:** 1 MOE Key Laboratory of Environment Remediation and Ecological Health, College of Environmental and Resource Sciences, Zhejiang University, Hangzhou 310058, China; 2 Key Laboratory of Subtropical Soil Science and Plant Nutrition of Zhejiang Province, College of Environmental and Resource Sciences, Zhejiang University, Hangzhou 310058, PR China; 3 Montverde Academy, Shanghai, 201702, China

## Abstract

Inhibition of root elongation is one of the most distinct symptoms of aluminium (Al) toxicity. Although putrescine (Put) has been identified as an important signaling molecule involved in Al tolerance, it is yet unknown how Put mitigates Al-induced root inhibition. Here, the possible mechanism was investigated by using two wheat genotypes differing in Al resistance: Al-tolerant Xi Aimai-1 and Al-sensitive Yangmai-5. Aluminium caused more root inhibition in Yangmai-5 and increased ethylene production at the root apices compared to Xi Aimai-1, whereas the effects were significantly reversed by ethylene biosynthesis inhibitors. The simultaneous exposure of wheat seedlings to Al and ethylene donor, ethephon, or ethylene biosynthesis precursor, 1-aminocyclopropane-1-carboxylic acid (ACC), increased ethylene production and aggravated root inhibition, which was more pronounced in Xi Aimai-1. In contrast, Put treatment decreased ethylene production and alleviated Al-induced root inhibition in both genotypes, and the effects were more conspicuous in Yangmai-5. Furthermore, our results indicated that Al-induced ethylene production was mediated by ACC synthase (ACS) and ACC oxidase, and that Put decreased ethylene production by inhibiting ACS. Altogether, these findings indicate that ethylene is involved in Al-induced root inhibition and this process could be alleviated by Put through inhibiting ACS activity.

Aluminium (Al) toxicity is a major constraint limiting crop growth and yield on acid soils, which occupy approximately 50% of the world’s potentially arable land^1^,^2^. Most Al exists in soils in non-toxic complexed forms; however, when soil pH drops below 5.0, phytotoxic forms of Al as hexaaquaaluminium [Al(H_2_O_2_)_6_]^3+^, or Al^3+^ ions may appear^3^. Low concentrations of Al rapidly inhibit root growth and function, subsequently leads to poor nutrient acquisition and reduced crop production^4^,^5^. Because Al is such a reactive element, a number of possible mechanisms for Al toxicity have been proposed. For example, Al may interact with multiple root cell sites, including the cell wall, plasma membrane, and symplasm, or it may interact with intracellular components, such as enzymes and proteins, which lead to the disruption of their functions^4^,^6^,^7^. Aluminium may also interfere with signal cascades in plants, such as cytosolic Ca^2+^ and 1,4,5-trisphosphate^8^,^9^. Plants have numerous strategies to withstand Al stress, among which the most well-characterised mechanism is Al exclusion from the root tips based on root exudation of organic acid^2^. Recently, genes involved in the Al-activated organic acid exudation have been identified in several plant species^2^,^3^. For example, *TaALMT1* (*Triticum aestivum* Al-activated malate transporter), which underpins the Al-induced wheat root malate exudation, has been identified as the major gene conferring Al resistance in wheat^10^. Although extensive progresses have been made during the past few years, the mechanisms of Al toxicity and tolerance remain elusive.

Ethylene, a gaseous plant hormone, is gradually becoming established as a vital co-regulator of plant growth and development under optimal and stressful conditions^11^,^12^. Rapidly increased ethylene production has frequently been observed in plant roots under Al stress^13^,^14^,^15^. Previous studies using ethylene synthesis inhibitors or ethylene-insensitive mutants demonstrated that the rapidly produced ethylene contributes to Al-induced root inhibition and, thus, relate to Al sensitivity, as demonstrated in *Lotus japonicas*, *Arabidopsis*, *Glycine max*^13^,^16^,^17^. In other plant species, however, it has been reported that enhanced ethylene production did not play a role in Al toxicity, for example in the roots of maize^18^. It is possible that the discrepancy between the studies is related to different plant species used and the kinetics of ethylene production. In plants, ethylene is synthesized from *S*-adenosylmethionine (SAM) and 1-aminocylopropane-1carboxylic acid (ACC), catalysed by ACC synthase (ACS) and ACC oxidase (ACO), respectively^19^. Although the cellular ethylene biosynthesis in higher plants is under strict metabolic regulation, the enzymes may change to some extent in response to abiotic stress^20^. Down-regulation of ethylene production through manipulating its biosynthesis enzymes has been considered as an essential strategy to enhance Al tolerance of crops, for example, in *Medicago*^21^. However, the mechanism and/or signal molecules involved in the modulation of Al-induced ethylene biosynthesis are largely unknown.

Putrescine (Put) is an essential signaling molecule involved in modulating plant resistance to Al stress^22^. For instance, exogenous Put promoted root growth under Al stress in saffron plants^23^, whereas Put biosynthesis inhibitors exacerbated the effects of Al in red kidney bean plants^24^. Currently available data also suggest that Put may interfere with ethylene biosynthesis or signaling transductions under stress conditions^25^,^26^. Hyodo and Tanaka^27^ found that Put suppressed ethylene production in a non-competitive manner. Further studies show that under osmotic stress, Put decreased stress-induced ethylene production through reducing the level of reactive oxygen species^26^. Since both of Put and ethylene have been implicated in the regulation of Al-induced root elongation inhibition, it is reasonable to assume that Put may alleviate Al-induced root inhibition, and subsequently Al toxicity through a mechanism of modulating ethylene production.

In this study, the above hypothesis was addressed by using pharmacological agents and two wheat genotypes differing in Al tolerance (Al-sensitive, Yangmai-5; Al-tolerant, Xi Aimai-1). We found that the differential Al sensitivity between the wheat genotypes was associated with their different ethylene production capacities, and that Put promoted root growth under Al stress by inhibiting ACS-mediated ethylene production.

## Results

### Putrescine alleviates Al-induced inhibition of root elongation

**Figure 1:**
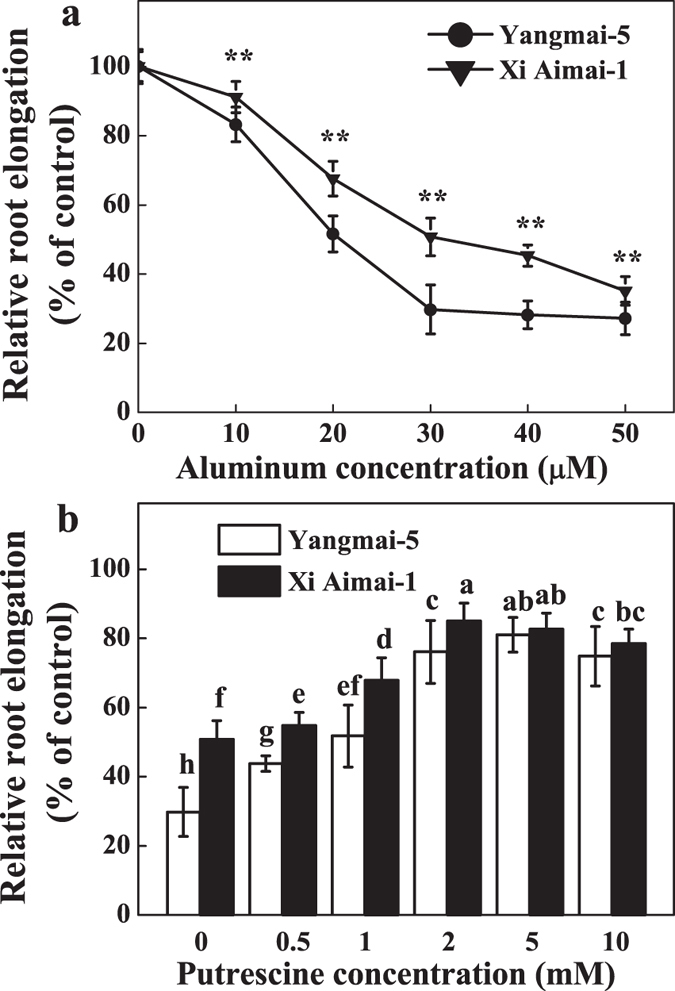
Root growth was measured. (**a**) Effects of increasing AlCl_3_ concentrations (0 ~ 50 μM) on root elongation of wheat seedlings. The primary root lengths were measured after the seedlings were exposed to Al for 24 h. (**b**) Effect of Put concentrations (0 ~ 10 mM) on Al-induced root inhibition of wheat seedlings. Seedlings were treated with Put at the indicated concentrations in the presence of 30 μM Al for 24 h. The control solution contained 0.5 mM CaCl_2_, pH 4.3 ± 0.1. Data are presented as the relative root elongation (RRE) compared to the control (CK) values. All values are means ± SD (n = 20). ** indicates a significant difference at *P* < 0.01. Columns with different letters are significantly different at *P* < 0.05. “n” refers to the number of independent experiments, same as follows.

**Figure 2:**
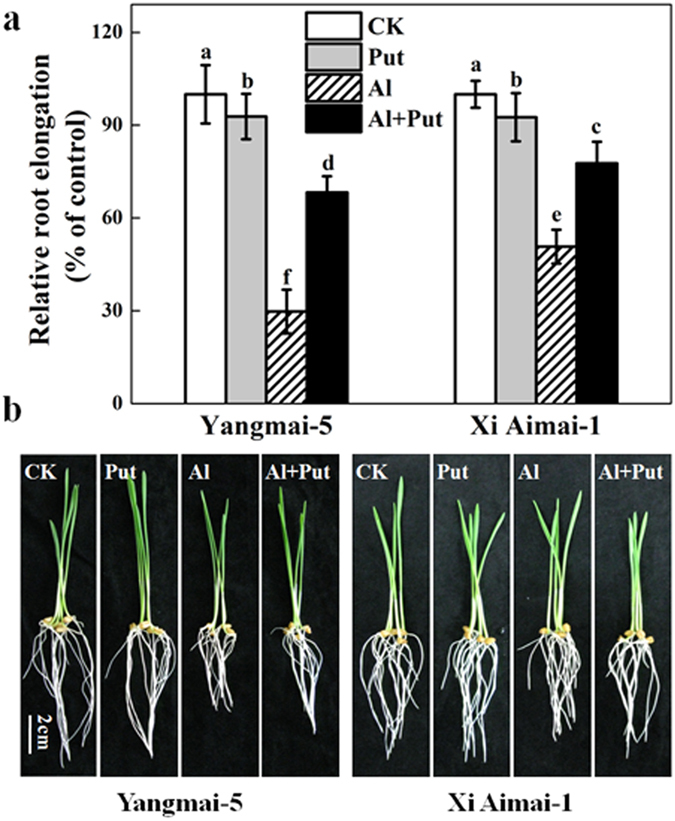
Effect of Put on Al-induced root inhibition of wheat seedlings. The 3-d-old seedlings were treated with 2 mM Put in the absence or presence of 30 μM Al for 24 h. (**a**) Root elongation. RRE was expressed relative to root elongation in the control solutions, which contained 0.5 mM CaCl_2_ (pH 4.3 ± 0.1). The values shown are means ± SD (n = 20). Columns with different letters are significantly different at *P* < 0.05. (**b**) Corresponding photographs. Bar, 2 cm.

Root elongation inhibition increased as the Al concentration rose (0, 10, 20, 30, 40, and 50 μM) in both genotypes. Al resistance, as determined by the RRE values, was more pronounced in Xi Aimai-1 than in Yangmai-5. Exposure to 30 μM AlCl_3_ resulted in the largest RRE difference between Yangmai-5 (30%) and Xi Aimai-1 (51%) (Fig. 1a). Thus, 30 μM Al was used in this study. Experiments on the effects of 0.5, 1, 2, 5, and 10 mM Put and 30 μM Al on root elongation were conducted in order to investigate whether Al-induced inhibition of root elongation can be alleviated by Put. A dose-dependent alleviation of Put on Al-induced root inhibition was observed at all concentrations tested. The 2 mM Put treatment had the most significant effect, and the amelioration was more efficient in Yangmai-5 than in Xi Aimai-1 (Fig. 1b). However, in the Al-free control, the 2 mM Put treatment slightly inhibited root elongation in both genotypes (Fig. 2a) (two-way ANOVA interaction, *P* < 0.001 for genotype, treatment and interactions). An ameliorating response by Put against Al-induced root inhibition was also morphologically observed (Fig. 2b).

### Effects of ethylene and Put on Al-induced inhibition of root elongation

**Figure 3:**
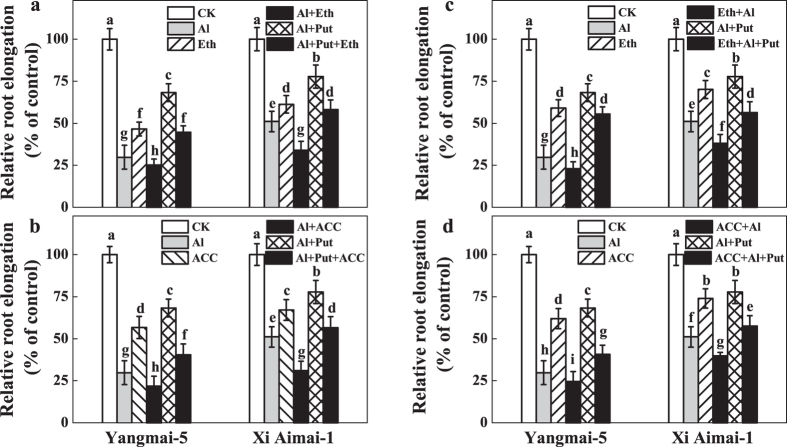
Effects of Al, ethylene-releasing substance ethephon (Eth), and ethylene biosynthesis precursor 1-aminocylopropane-1carboxylic acid (ACC) on wheat plant root growth. Seedlings were treated with 30 μM Eth (**a**), 10 μM ACC (**b**) in the absence or presence of Al or Al + Put for 24 h, or pretreated with 30 μM Eth (**c**) or 10 μM ACC (**d**) for 3 h and then exposed to Al for 24 h. RRE was expressed relative to root elongation in control solutions containing 0.5 mM CaCl_2_ (pH 4.3 ± 0.1). The values shown are means ± SD (n = 20). Columns with different letters are significantly different at *P* < 0.05.

**Figure 4:**
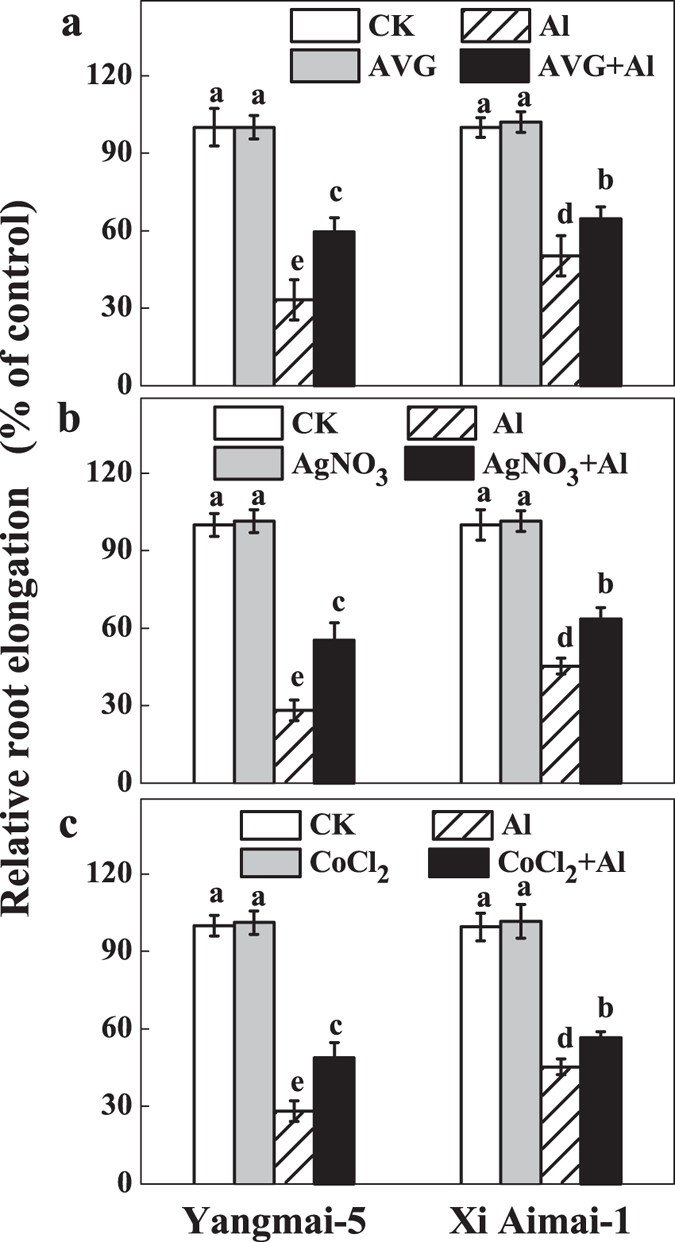
Effects of ethylene synthesis inhibitors (AVG, AgNO_3_, and CoCl_2_) on Al-induced root inhibition of wheat seedlings. The 3-d-old seedlings were pretreated with 1 μM AVG (**a**), 10 μM AgNO_3_ (**b**), or 10 μM CoCl_2_ (**c**) for 3 h and then exposed to Al for 24 h. RRE was expressed relative to root elongation in control solutions containing 0.5 mM CaCl_2_ (pH 4.3 ± 0.1). The values shown are means ± SD (n = 20). Columns with different letters are significantly different at *P* < 0.05.

To confirm the role of ethylene in Al-induced root growth inhibition, further experiments were performed that manipulated ethylene levels or ethylene signaling by using ethylene biosynthesis inhibitors (AVG and CoCl_2_) or an ethylene perception blocker (AgNO_3_), ethephon, and ACC and Put separately or simultaneously. As shown in Fig. 3, treatment with 30 μM ethephon or 10 μM ACC inhibited root elongation and aggravated Al-induced root inhibition in both wheat genotypes, but the aggravation was more serious in Xi Aimai-1 (two-way ANOVA interaction, *P* < 0.001 for genotype, treatment and interactions). In contrast, pretreatment with 1 μM AVG, 10 μM CoCl_2_, or 10 μM AgNO_3_, which had little effect on root elongation under the control conditions, significantly alleviated root inhibition under Al stress by 26%, 21%, and 27% for Yangmai-5, and 14%, 11%, and 18% for Xi Aimai-1, respectively (Fig. 4) (two-way ANOVA interaction, *P* < 0.001 for genotype, treatment and interactions). Interestingly, Put treatment alone also alleviated Al-induced root inhibition by 38% and 27% in Yangmai-5 and Xi Aimai-1, respectively. In addition, application of ethephon/ACC partially reversed the alleviation of root inhibition by Put under Al stress in both wheat genotypes (Fig. 3). To avoid a direct interaction between ethephon/ACC and Put, an ethephon/ACC pretreatment experiment was also conducted and similar effects to roots treated with ethephon/ACC, and Al or Al + Put simultaneously, were observed (Fig. 3c,d).

### Effects of Al and Put on ethylene production in the root tips of wheat seedlings

**Figure 5:**
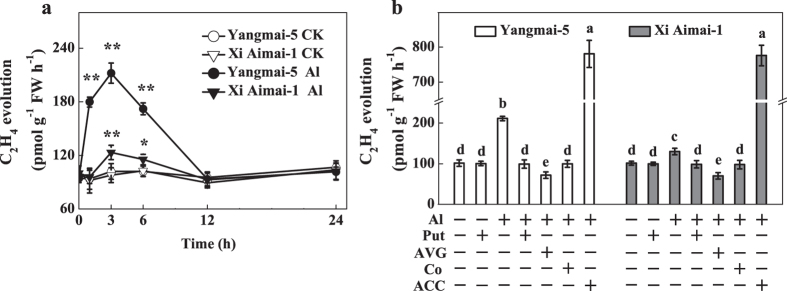
Ethylene production in root tips of wheat plants. (**a**) Time-course for ethylene production from root apexes of wheat seedlings exposed to 30 μM AlCl_3_. (**b**) Effects of Al or Put alone, Al + Put, ethylene synthesis or perception inhibitors (AVG, AgNO_3_, and CoCl_2_), or ethylene precursor (ACC) on ethylene production. Roots of 3-d-old seedlings were pretreated with 1 μM AVG or 10 μM CoCl_2_ for 3 h and then exposed to Al for another 3 h, or directly treated with Al + Put for 3 h. Root apexes were excised to determine ethylene production. Data are means ± SD (n = 3). * and ** indicate significant differences between CK and Al treatments at *P* < 0.05 and *P* < 0.01, respectively. Columns with different letters are significantly different at *P* < 0.05.

The effect of Al^3+^ on ethylene evolution from the root tips of wheat plants was analyzed to further clarify whether Al-induced root inhibition is related to the induction of ethylene production. The roots of the control samples represented the basal ethylene levels. After Al treatment, significant differences in ethylene production were observed in the root tips of the two wheat genotypes (Fig. 5). A significant burst of ethylene was observed in Al-sensitive Yangmai-5 after 1.5 h Al exposure and the maximum ethylene content was reached at 3 h, but then production declined to a relatively steady level after 12 h. However, in the Al-tolerant Xi Aimai-1, ethylene had only slightly increased after 3 h Al treatment and the level was 1.7-fold lower than that in Yangmai-5 (Fig. 5a). Treatment with exogenous Put significantly reduced Al-induced ethylene production by approximately 106% and 36% in Yangmai-5 and Xi Aimai-1, respectively. A similar effect was also observed in the CoCl_2_ and AVG treatments (Fig. 5b), which suggested that the ameliorating effects of CoCl_2_ and AVG on Al-induced root inhibition were associated with Al-elicited ethylene biosynthesis. This effect might also be responsible for the improved root growth induced by Put.

### Effects of Put on the activities of ethylene synthesis enzymes

**Figure 6:**
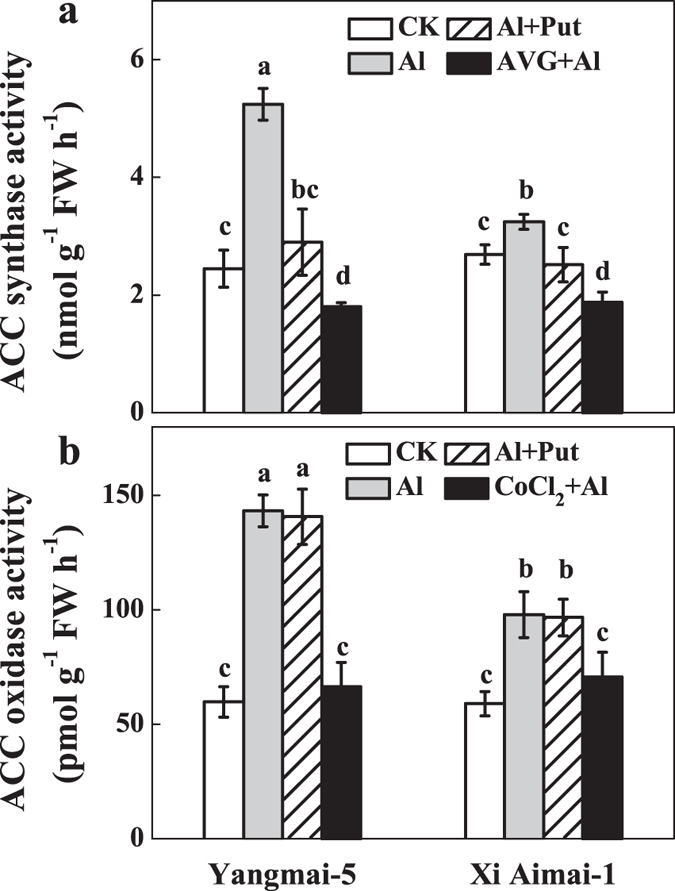
Effects of Put and ethylene synthesis inhibitors (AVG and CoCl_2_, respectively) on ACC synthase (ACS) (a) and ACC oxidase activity (b) in root apexes of wheat seedlings. The 3-d-old seedlings were pretreated with 1 μM AVG (an ACS inhibitor) or 10 μM CoCl_2_ (an ACO inhibitor) for 3 h, and then exposed to Al or Al + Put for another 3 h. Then, the root apexes were collected for assaying ACS and ACO activity. Data are means ± SD (n = 3). Columns with different letters are significantly different at *P* < 0.05.

ACS and ACO are two key enzymes responsible for ethylene production in plants^28^. We measured the ACS and ACO activities to determine the possible mechanism underlying Put decreased ethylene production in the root tips of wheat. As shown in Fig. 6, the activities of both ACS and ACO increased in the root tips of the two wheat genotypes, with the former increasing much more than the latter. Furthermore, both enzyme activities in Yangmai-5 were much higher than in Xi Aimai-1 (Fig. 6). Application of Put significantly reduced ACS activity, but it had little effect on ACO activity, which suggested that Put may reverse Al-induced ethylene production by inhibiting the activity of ACS.

### Effects of Put on the content of ethylene precursor ACC

**Figure 7:**
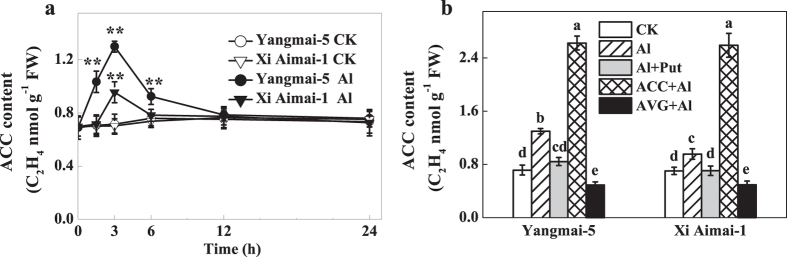
ACC content in root tips of wheat plants. (**a**) Time-course for ACC content in wheat root tips upon exposure to 30 μM AlCl_3_. (**b**) Effect of Put, ACC, and AVG on ACC content in wheat root tips under Al stress. The 3-d-old seedlings were pretreated with 10 μM ACC or 1 μM AVG for 3 h, and then exposed to Al for 3 h, or they were treated with Al + Put for 3 h. Root apexes were collected to determine the ACC content. Data are means ± SD (n = 3). ** indicate significant differences between CK and Al treatments at *P* < 0.01. Columns with different letters are significantly different at *P* < 0.05.

Changes in ACC levels were analyzed to further confirm whether the Put-reduced ethylene production in wheat roots was related to ACC biosynthesis. Figure 7a shows that the increases in ACC content caused by Al were similar to the ethylene production changes seen in wheat plants, whereas the application of AVG or Put suppressed the increase (Fig. 7b). In addition, ACC application further increased endogenous ACC content in both genotypes. It has been reported that ACC per se has a short-term (a few hours) influence on root cell elongation^29^. In this study, ACC application further enhanced Al-induced ethylene production (Fig. 5b) and the inhibitory effect of ACC on RRE was mostly alleviated in the presence of AgNO_3_ (Fig. S1), which suggested that ACC exerts its effect mainly after conversion to ethylene.

## Discussion

Plant responses to Al toxicity are expressed by a myriad of changes in the physiological processes, and also involve various signal components^7^,^23^. As a crucial signaling molecule, Put plays essential roles for plant performance under stress conditions^30^,^31^. In the present study, Put was found to significantly alleviate the growth inhibition of wheat plants subjected Al stress. Al tolerance in wheat plants was shown to be highly associated with the *TaALMT1*-mediated malate exudation from the root tips^2^,^10^. However, application of Put had little effect on the expression of *TaALMT1* in the root tips of both wheat genotypes under Al stress (Fig. S2), suggesting that the Put-related improved Al tolerance might not be associated with *TaALMT1*-mediated malate efflux. Furthermore, in our previous study, we showed that Put did not affect Al-induced malate secretion in wheat plants^34^, providing wfurther evidence for the above assumption. In this study, we found that Put alleviates root growth inhibition through inhibiting ethylene biosynthesis, which is rapidly produced and behaved as a negative regulator of root elongation.

Inhibition of root elongation is the primary manifestation of Al toxicity, and RRE has been frequently used to assess Al resistance in plants^32^,^33^. In this study, We found that inhibition of root elongation increased with increasing external Al concentrations in both Al-sensitive (Yangmai-5) and Al-tolerant (Xi Aimai-1) wheat genotypes, with the effect being much less pronounced in the latter (Fig. 1a). Recently, there has been some experimental evidence supporting the involvement of Put in plant tolerance to Al stress. Our previous study revealed a marked Put accumulation in the root tips of wheat plants^34^. Here, exogenous Put significantly alleviated Al-induced root inhibition in both wheat genotypes, and the effect was more pronounced in Yangmai-5 than in Xi Aimai-1 (Fig. 2). Similar results were observed in red kidney bean^22^,^24^, saffron^23^, and Salvinia^35^, in which exogenous Put alleviated while Put biosynthesis inhibitors exaggerated Al toxicity.

Ethylene is a plant hormone that plays prominent roles in modulating root growth^36^. Recent studies suggested that ethylene may also regulate root response to Al in plants^37^,^38^. In our study, a similar effect on root growth in wheat plants to Al stress was caused by an ethylene-releasing substance (ethephon) and an ethylene precursor (ACC) (Fig. 3). We also observed that Al caused a rapid production of ethylene in the root tips of Yangmai-5 after 1.5 h. The Al-elicited ethylene production reached a maximum after 3 h exposure to Al, and the level was significantly higher in Yangmai-5 than in Xi Aimai-1 (Fig. 5). The difference in Al content may not be responsible for the variation in ethylene production since there was no significant difference in Al content in the root tips of the two wheat genotypes before 6 h (Fig. S3). This suggests that ethylene may be involved in Al-induced root inhibition of wheat seedlings and that the genotypic variations in ethylene production may contribute to their difference in Al sensitivity. The observation that application of ACC, which increased ethylene production in the root tips of both wheat genotypes (Fig. 5b), and aggravated Al-induced root inhibition more in Xi Aimai-1 than in Yangmai-5 (Fig. 3b) is in line with this proposition. This was further supported by the application of ethylene biosynthesis (AVG and CoCl_2_) or ethylene perception (AgNO_3_) antagonists, which markedly reduced Al-induced ethylene production (Fig. 5b) or blocked its action and efficiently promoted root growth of both wheat genotypes under Al stress (Figs 3 and 4). Therefore, our results indicated that the ethylene production induced by Al occurred very rapidly and might be responsible for Al-induced root inhibition.

Many studies have suggested that Put and ethylene have opposite effects in many different plant processes and abiotic stress responses^39^,^40^,^41^. Put has also been reported to interact with ethylene biosynthesis under various stress conditions^27^,^42^. However, the interaction between Put and ethylene under Al stress remains undetermined. Our results showed that exogenous Put inhibited Al-induced ethylene production in the roots of wheat seedling subjected to Al stress, which was similar to the effects of AVG or CoCl_2_ (Fig. 5b). Importantly, these changes were related to the recovery from root inhibition caused by Al exposure (Figs 2 and 3). Furthermore, pretreatment with ethephon or ACC was able to counteract the Put-induced alleviation of root inhibition by Al stress (Fig. 3c,d). These results clearly suggest that, at least partially, Put improved root growth by inhibiting ethylene production under Al stress. The fact that the ameliorative effect of Put was more prominent in Yangmai-5, which produced higher levels of ethylene than Xi Aimai-1, further proved this conjecture. In terrestrial plants, the ethylene and polyamine pathways are generally considered to be competitive^43^,^44^. Although exogenous Put slightly increased spermidine (Spd) content (data not shown) and decreased ethylene production, the two pathways were not strictly antagonistic in our study because the addition of AVG, which inhibits the conversion of SAM (the common precursor of Spd and ethylene) to ethylene, did not enhance polyamine biosynthesis (Fig. S4). A better explanation could be that the availability of SAM *in vivo* is not rate limiting during the biosynthesis of either ethylene or Spd, and that both pathways could run simultaneously^45^,^46^. Evaluation of the potential sources of ethylene revealed that Al-induced root inhibition might be due to the increase in both ACS and ACO activities (Fig. 6). However, ACS and ACO have been identified as two sites where Put can affect ethylene biosynthesis^25^,^47^. The effects of Put on the activities of ACS and ACO, and ACC content were examined to further unravel how Put decreases ethylene production under Al stress. Our results suggested that Put inhibited ethylene production by directly suppressing ACS activity at the step where SAM was converted to ACC (Figs 6a and 7).

**Figure 8:**
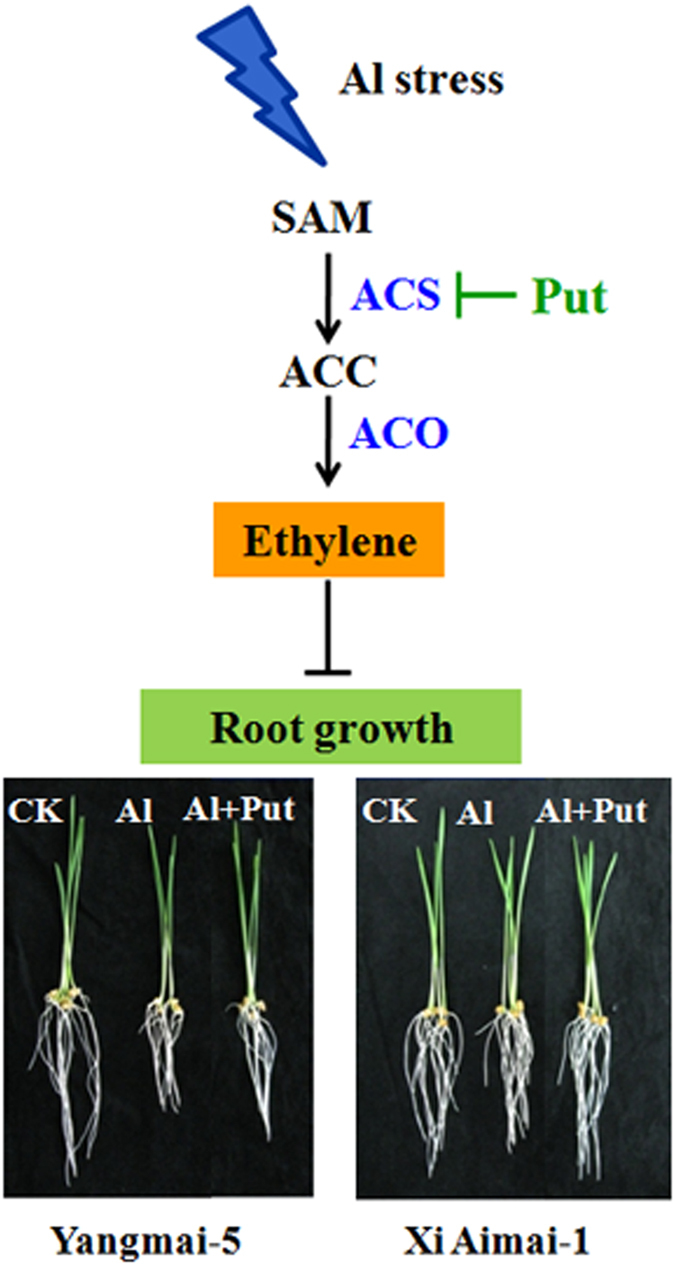
Proposed model of how Put alleviates Al-induced root inhibition. Al activates ACS and ACO, which promote ethylene biosynthesis and inhibit root elongation, whereas Put suppresses ACS, which blocks the beginning of ethylene biosynthesis and thus reduces Al-elicited ethylene production, leading to increased root growth.

In summary, our study reveals the protective role of Put on Al-induced root inhibition of wheat plants. We also demonstrated that ethylene may be involved in Al-induced root inhibition and the different ethylene production profiles may be due to the differential Al sensitivity between the two wheat genotypes. Most importantly, Put application reduced ACS activity, and thus ethylene production, which may explain how Put alleviated root inhibition under Al tress. Our results not only suggested a potential mechanism for Al-induced root inhibition, but also provided a possible explanation for the function of Put in plants. We therefore proposed a simple model to explain the integration between Put and ethylene under Al stress (Fig. 8).

## Materials and Methods

### Plant materials

Seeds of two wheat genotypes Yangmai-5 and Xi Aimai-1, which are classified as Al-sensitive and Al-tolerant genotypes previously^48^,^49^, were used in this study. The seeds were surface sterilized with 1% NaClO for 20 min and rinsed in distilled water overnight. After germination in the dark for 12 h at 25 °C, the seeds were transplanted to plastic screens floating on 0.5 mM CaCl_2_ solution (pH 4.3 ± 0.1) in a growth chamber under a 12 h/25 °C day and 12 h/22 °C night regime, a light intensity of 300 μmol m^−2^ s^−1^, and a relative humidity of 70%. The solutions were renewed daily. The seedlings were exposed to the various treatments when the average root length was about 45 mm. After that, the roots at selected time points after treatment were washed with distilled water several times and the root apexes (~10 mm) were excised for further analysis.

### Measurement of root elongation

To study the effect of ethylene, aminoethoxyvinylglycine (AVG), CoCl_2_, and AgNO_3_ on root elongation in the absence and presence of Al, 3-d-old seedlings were treated with 30 μM ethephon (an ethylene-releasing substance) or 10 μM ACC (an ethylene precursor), 10 μM AVG (an inhibitor of ACS), 10 μM CoCl_2_ (an inhibitor of ACO) and 10 μM AgNO_3_ (an antagonist of ethylene perception) together with 30 μM AlCl_3_ for 24 h or pretreated with above reagents for 3 h and then exposed to 30 μM AlCl_3_ for another 24 h. Elongation of the primary root was measured with a ruler before and after treatment. Relative root elongation (RRE, %) was calculated as the percentage of the root elongated by various treatments normalised over the Al-free control. Values of RRE were given as means ± SD of at least twenty independent measurements. All experiments were repeated at least three times.

### Measurement of ethylene production

Ethylene production was determined according to Sun *et al.*^13^, with slight modifications. Briefly, root tips (~10 mm in length) were excised. To minimize the wounding effect, the excised root apexes were put into 6 mL gas-tight vials for 0.5 h before they were sealed. After incubation at room temperature for 2 h in the dark, a 1 mL gas sample of the headspace was taken from the vials with a syringe. The ethylene concentration in the samples was measured by injecting the sample into a gas chromatograph (Agilent 7890 A, USA) equipped with a Porapak Q column and a flame ionization detector (FID).

### Extraction and analysis of ACC

The ACC contents in wheat roots were extracted and analyzed according to Wang *et al.*^50^, with slight modifications. Root apexes (~10 mm) were pulverized in liquid nitrogen and homogenized in 1 mL of 80% ethanol at 55 °C for 10 min. The slurry was then centrifuged at 10,000 *g* for 10 min. The supernatant was saved and the extraction was repeated twice. The final samples were combined and evaporated to dryness in vacuum at 55 °C, and then resuspended with 2.5 mL distilled water. The extracted ACC in the aqueous part was quantified indirectly by converting ACC to ethylene. The ethylene evolved from ACC was assayed using gas chromatography as described above.

### Assays for ACS and ACO activity

The ACS and ACO activity was determined as described by Woeste *et al.*^51^ and Tian *et al.*^36^. For ACS determination, root apexes (~10 mm) were ground with liquid nitrogen, and resuspended in 1.5 mL extract buffer containing 200 mM phosphate buffer (pH 8.0), 10 μM pyridoxal phosphate, 1 mM EDTA, 2 mM phenylmethylsulfonyl fluoride (PMSF), and 5 mM dithiothreitol (DTT). The extract was centrifuged at 15,000 *g* for 15 min at 4 °C. A 600 μL aliquot of the supernatant was transferred to a 6 mL gas-tight vial containing 200 μL 5 mM *S*-(5′-Adenosyl)-*L*-methionine (Sigma-Aldrich). The reaction was carried out for 1 h at 22 °C. The ACC formed was converted to ethylene by adding 200 μL of a 1:1 (v:v) mixture containing saturated NaOH:bleach. The reaction vials were quickly sealed and kept in ice for 20 min. Ethylene produced in the head space was determined as described above.

To determine ACO activity, root apexes (~10 mm) were ground with liquid nitrogen and resuspended in 1.5 mL extract buffer containing 0.1 M Tris-HCl (pH 7.2), 10% (w:v) glycerol, 30 mM sodium ascorbate, and 5% (w:v) polyvinyl polypyrolidine (PVPP). After centrifuging at 15,000 *g* for 20 min at 4 °C, the ACO activity was assayed immediately by mixing 1 mL of the supernatant with 1.7 mL of extraction buffer (without PVPP), 50 μM FeSO_4_, and 2 mM ACC. Then the mixture was incubated at 30 °C for 1 h. The ethylene produced in the head space was determined as described earlier.

### Statistical analysis

The data were subjected to analysis of variance (ANOVA), and the least significant difference (LSD) test was employed to determine differences among the treatments at *P* = 0.01 and *P* = 0.05 levels.

## Additional Information

**How to cite this article**: Yu, Y. *et al.* Inhibition of ethylene production by putrescine alleviates aluminium-induced root inhibition in wheat plants. *Sci. Rep.* **6**, 18888; doi: 10.1038/srep18888 (2016).

## Supplementary Material

Supplementary Information
