# Supplementary material for: Inhibition of ethylene production by putrescine alleviates aluminium-induced root inhibition in wheat plants
Source: Sci Rep. 2016 Jan 8;6:18888. doi: 10.1038/srep18888 (PMC4705537; doi:10.1038/srep18888)
Supplement: Supplementary Information [file srep18888-s1.pdf]

Supplementary Information

Title:

Inhibition of ethylene production by putrescine alleviates aluminium-induced root inhibition in wheat plants

Authors:

Yan Yu[1,a], Chongwei Jin[1,2,a], Chengliang Sun[1], Jinghong Wang[3], Yiquan Ye[1], Weiwei Zhou[1], Lingli Lu[1,2], Xianyong Lin[1,2*]

Institutions:

[1]MOE Key Laboratory of Environment Remediation and Ecological Health, College of Natural Resource and Environmental Sciences, Zhejiang University, Hangzhou 310058, China

[2]Key Laboratory of Subtropical Soil Science and Plant Nutrition of Zhejiang Province, College of Environmental and Resource Sciences, Zhejiang University, Hangzhou 310058, PR China

[3]Montverde Academy, Shanghai, 201702, China.

[a]these authors contributed equally to this work.

***Corresponding author**

Prof. Dr. Xianyong Lin

College of Natural Resource and Environmental Sciences,

Zhejiang University, Hangzhou 310058, China

Tel.: +86 571-88982476; fax: +86 571-86971395

E-mail address: xylin@zju.edu.cn

Materials and methods

Plant materials

Seeds from two winter wheat genotypes, Yangmai-5 (Al-sensitive) and Xi Aimai-1 (Al-tolerant), were used in this study. The seeds were surface sterilized with 1% NaClO for 20 min and rinsed in distilled water overnight. After germination in the dark for 12 h at 25°C, the seeds were transplanted to plastic screens floating on 0.5 mM $CaCl_2$ solution (pH 4.3 ± 0.1) in a growth chamber under a 12 h/25°C day and 12 h/22°C night regime, a light intensity of 300 μmol m^{-2} s^{-1}, and a relative humidity of 70%. The solutions were renewed daily. The seedlings were exposed to the various treatments when the average root length was about 45 mm. After that, the roots at selected time points after treatment were washed with distilled water several times and the root apexes (~10 mm) were excised for further analysis. Each experiment was repeated at least three times. Elongation of the primary root was measured with a ruler before and after treatment. Relative root elongation (RRE) was defined as the percentage root elongation in the treated solutions compared to those in the control solution.

Al content determination in root tips

For Al content determination in root tips, excised root tips were directly suspended in 10 mL of 2 M HCl with occasional shaking for 24 h. The Al concentrations in the HCl solution were determined by inductively coupled plasma mass spectrometer (ICP/MS) Agilent 7500A (Agilent, Palo Alto, CA, USA) according to Xu et al.[1].

Analysis of *TaALMT1* gene expression

The expression of *TaALMT1* was determined by real-time PCR. Briefly, RNA was isolated from 100 mg fresh-weight wheat seedling root tips (0-10 mm) using Trizol reagent according to the manufacturer's protocol (Life Technologies, Rockville, MD, USA). One micrograms of the RNA from each sample was reverse-transcribed into first-strand cDNA with PrimeScript RT reagent Kit (Takara, Dalian, Liaoning, China) according to the manufacture's protocol. The first-strand cDNA was used for SYBR Green-monitored qRT-PCR (Takara). The qRT-PCR analysis was performed using the MJ Option™ 2Real-Time PCR System (MJ Research™). The primers used for real-time PCR analysis for *TaALMT1* were those used by Sasaki et al.3: 5'-AAGAGCGTCCTTAATTCG-3' and 5'-CCTTACATGATAGCTCAGGG-3', and those for the housekeeping gene *TaActin* was 5'-CTATCCTTCGTTTGGACCTT-3' and 5'-GCGAGCTTCTCCTTTATGT-3'. The expression of *TaALMT1* was calculated from the relative expression levels of *TaALMT1* and the expression levels of the reference gene *TaActin* using arbitrary units. Three biological and three technological repeats were performed in RT-PCR. The relative expression level was analysed by the comparative C_T method.

Determination of polyamines by high performance liquid chromatography (HPLC)

The concentrations of free polyamines were estimated following the methods described by Flores and Galston[2] with slight modifications. Briefly, root apexes were homogenized in 5% cold perchloric acid (PCA), and incubated on ice for 1 h, and

then centrifuged at 12,000*g* for 20 min at 4°C. The pellet was extracted twice with 5% PCA. Then, the supernatants were collected and derivatized using the benzoylation method. A 150 μL aliquot of PCA extracts was mixed with 1 mL of 2 M NaOH, followed by addition of 10 μL benzoyl chloride, vortexing for 20 s, and incubation for 25 min at 37°C. Then, 2 mL of saturated NaCl was added immediately to stop the reaction. Benzoyl polyamine derivatives were extracted with 2 mL of diethyl ether. After centrifugation at 1500*g* for 5 min, 1 mL of the ether phase was collected, evaporated to dryness, and dissolved in 200 μL of methanol. After filteration through 0.25 μm nylon membranes, 10 μL of the methanol solution of benzoyl polyamines was analyzed using a HPLC system (Agilent 1200, USA) and UV detector under the following conditions: 4.6 mm × 150 mm Elipse XDB-C18 reverse-phase column (Agilent, USA); particle size, 5 μm; column temperature, 30°C; mobile phase, 65% methanol; flow rate, 0.6 mL min^{-1}; and detection wavelength, 254 nm. Three polyamine standards of Put, Spd, and Spm in the form of hydrochlorides (Sigma) were benzoylated simultaneously.

Supplementary Fig. S1

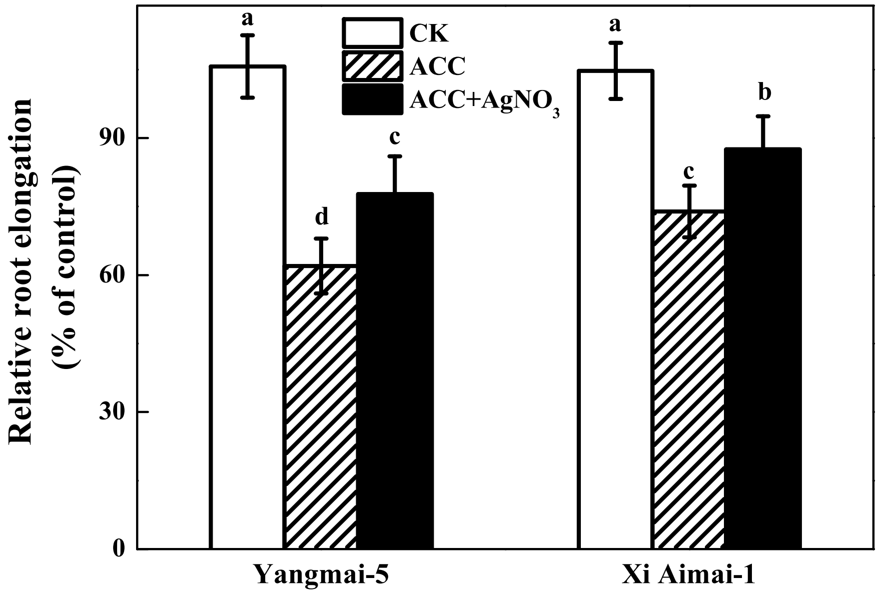

Supplementary Fig. S1 Effects of Ag^{+} on ACC-induced inhibition of root elongation. The 3-d-old seedlings were pretreated with 10 μM ACC or 10 μM ACC plus 10 μM $AgNO_3$ for 3 h and then cultured in solutions containing 0.5 mM $CaCl_2$ (pH 4.3 ± 0.1) for 24 h. RRE was expressed relative to root elongation in control solutions containing 0.5 mM $CaCl_2$ (pH 4.3 ± 0.1). The values shown are means ± SD (n = 20). Columns with different letters are significantly different at $P < 0.05$.

Supplementary Fig. S2

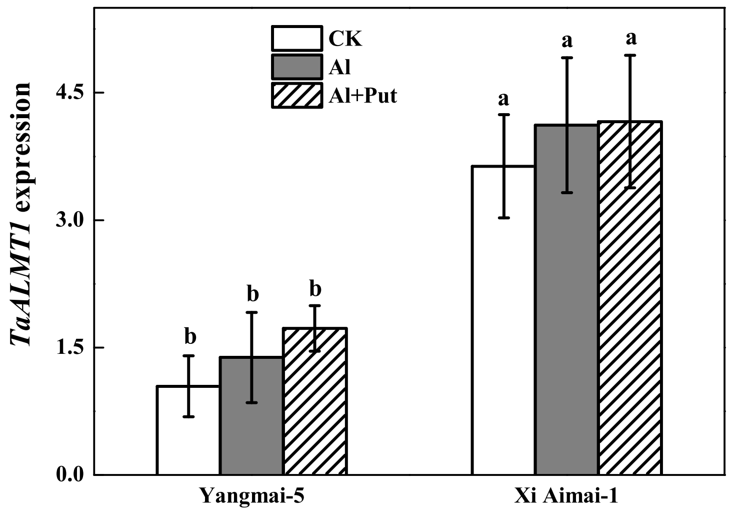

Supplementary Fig. S2 Effects of Al and Put on *TaALMT1* expression of wheat seedlings. Root tips were collected after 24 h exposure and the expression of *TaALMT1* in the root tips was determined using qRT-PCR. Means $\pm$ SD (n = 3). Columns with different letters are significantly different between treatments at $P <$ 0.05.

Supplementary Fig. S3

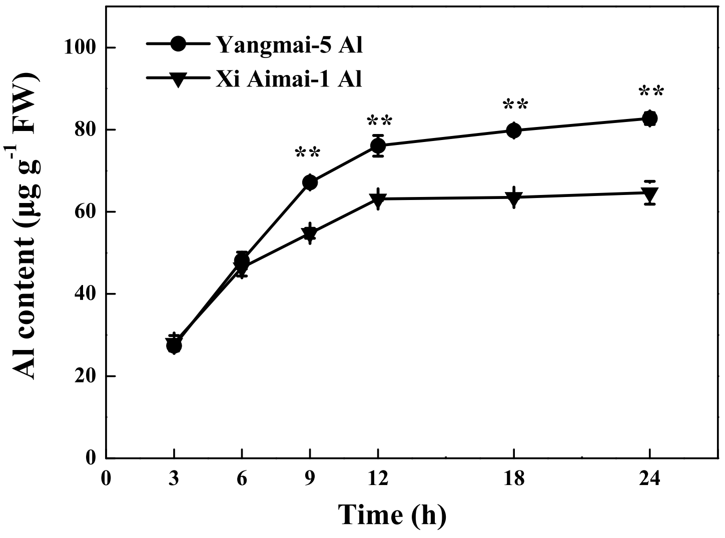

Supplementary Fig. S3 Time course for Al content in root tips of wheat seedlings over 24 h of Al treatment. Roots of 3-d-old seedlings treated with 30 μM Al were harvested at indicated time, and washed with deionized water. Data are means ± SD of

three replicates. ** indicate significant differences among treatments at $P < 0.01$.

Supplementary Fig. S4

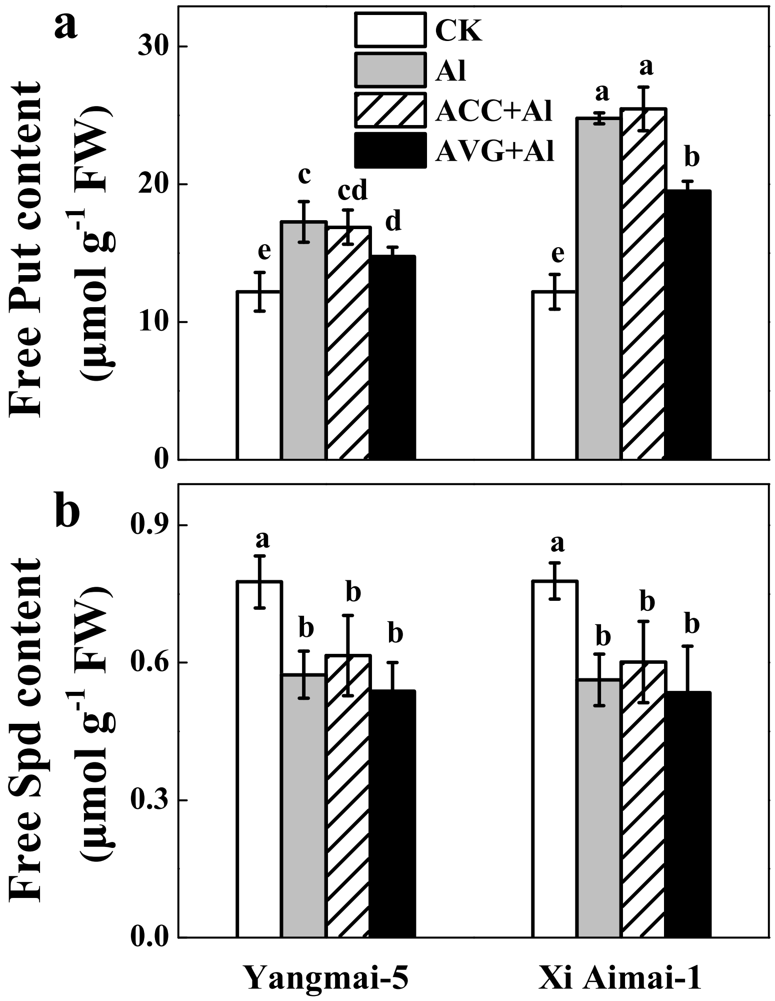

Supplementary Fig. S4 Effects of ethylene biosynthesis precursor (ACC) and inhibitor (AVG) on Put (A) and Spd (B) content in wheat seedlings roots under Al stress. Roots of 3-d-old seedlings were pretreated with 10 μM ACC or 1 μM AVG and then exposed to Al for 24 h. Root tips were excised to determine the Put (A) and Spd (B) contents. Data shown are means ± SD (n = 3). Columns with different letters are significantly different at $P < 0.05$.

References

1 Xu, F. J., Jin, C. W., Liu, W. J., Zhang, Y. S. & Lin, X. Y. Pretreatment with H_2O_2 alleviates aluminum-induced oxidative stress in wheat seedlings. *J. Integr. Plant Biol.* **53**, 44-53 (2011).

2 Flores, H. E. & Galston, A. W. Analysis of polyamines in higher plants by high performance liquid chromatography. *Plant Physiol.* **69**, 701-706 (1982).

3 Sasaki T. *et al*. Sequence upstream of the wheat (*Triticum aestivum* L.) *ALMT1* gene and its relationship to aluminum resistance. *Plant cell Physiol.* **10**, 1343-1354 (2006).
